# Correction: Khdary et al. Preparation of Cu/Sn-Organic Nano-Composite Catalysts for Potential Use in Hydrogen Evolution Reaction and Electrochemical Characterization. *Nanomaterials* 2023, *13*, 911

**DOI:** 10.3390/nano16040227

**Published:** 2026-02-10

**Authors:** Nezar H. Khdary, Gaber El Enany, Amani S. Almalki, Ahmed M. Alhassan, Abdullah Altamimi, Saeed Alshihri

**Affiliations:** 1 Institute of Materials Science, King Abdulaziz City for Science and Technology, Riyadh 11442, Saudi Arabia; assalmalki@kacst.edu.sa (A.S.A.); amalhassan@kacst.edu.sa (A.M.A.); aalhassan@kacst.edu.sa (A.A.); alshihri@kacst.edu.sa (S.A.); 2 Department of Physics, College of Science and Arts in Uglat Asugour, Qassim University, Buraydah 52571, Saudi Arabia; g.elenany@qu.edu.sa

In the original publication [1], reference 20 was retracted prior to this publication. Concerns were raised on this matter. The authors would like to replace the reference with the following one:

20. Jia, L.; Wang, J.; Zhang, B.; Ruan, Y.; Wan, H.; Ji, X.; Xu, K.; Dace, Z.; Miao, L.; Jiang, J. Mutually Beneficial Co_3_O_4_@MoS_2_ Heterostructures as Highly Efficient Bifunctional Catalyst for Electrochemical Overall-Water-Splitting. *J. Mater. Chem. A* **2018**, *6*, 2067–2072. https://doi.org/10.1039/C7TA10048E.

The authors state that the scientific conclusions are unaffected. This correction was approved by the Academic Editor. The original publication has also been updated.
